# Exploring the Protective Role of G6PD Deficiency in Aluminum Phosphide Poisoning: A Case Report and Review of the Literature

**DOI:** 10.7759/cureus.58888

**Published:** 2024-04-24

**Authors:** Rizwanullah, Yoalkris E Salcedo, Jayati Mukesh Mehta, Jubran Al Balushi, Mansi Khariwal, Netra Patel

**Affiliations:** 1 Internal Medicine, Hayatabad Medical Complex, Peshawar, PAK; 2 Surgery, Universidad Iberoamericana, Santo Domingo, DOM; 3 Epidemiology and Public Health, Icahn School of Medicine at Mount Sinai, New York, USA; 4 Medicine, University College Dublin, Dublin, IRL; 5 Medicine and Surgery, Richmond University Medical Center, New York, USA; 6 Medicine and Surgery, American University of Antigua, Osbourn, ATG

**Keywords:** literature review, case report, antidotes, survival outcomes, phosphine gas, protective effects, oxidative stress, g6pd deficiency, aluminium phosphide (alp) poisoning, wheat pil poisoning

## Abstract

Aluminum phosphide (ALP) poisoning poses a significant public health concern worldwide, with a high mortality rate and no established definitive treatment. This case report highlights a 30-year-old male with G6PD deficiency who ingested ALP tablets, presenting with jaundice and anemia. Despite the severity of ALP poisoning, the concurrent G6PD deficiency appeared to confer a protective effect, potentially mitigating complications. Laboratory investigations revealed characteristic findings, including unconjugated hyperbilirubinemia and normocytic hypochromic anemia. Treatment involved supportive measures and transfusion, leading to clinical improvement and discharge. The discussion focuses on the pathophysiology of G6PD deficiency and its protective role against ALP poisoning, supported by a literature review and experimental evidence. Moreover, potential therapeutic interventions targeting oxidative stress are discussed. This case underscores the importance of considering G6PD deficiency in ALP poisoning management and highlights avenues for further research into protective mechanisms and treatment strategies.

## Introduction

Aluminum phosphide (ALP), commonly known as rice/wheat pill, is readily available as an insecticide and rodenticide, contributing significantly to fatalities in numerous countries. The incidence of cases arriving at medical facilities with a history of ALP poisoning has substantially risen in recent years. Despite ongoing efforts, there is currently no established definitive treatment for this poisoning. The mortality rate associated with severe cases can be extremely high, reaching approximately 98-100% [1].

Ingestion of ALP can result in a diverse range of symptoms, including nausea, diarrhea, headache, abdominal pain, hypotension, congestive heart failure, myocarditis, and arrhythmia. Respiratory manifestations may include dyspnea, acute respiratory distress syndrome (ARDS), and pulmonary edema. Additionally, one of the less common manifestations of ALP poisoning is intravascular hemolysis [2].

Here, we present a case of wheat pills/ALP poisoning in an individual who also exhibited G6PD deficiency. Interestingly, the G6PD deficiency seemed to have acted protectively, potentially averting the onset of complications associated with ALP poisoning.

## Case presentation

A 30-year-old male patient, a driver by profession, with a history of depression, presented to the medical emergency department of Hayatabad Medical Complex, having ingested two tablets of wheat pills/ALP six days prior. He arrived at a local hospital with symptoms including dyspnea, cough, abdominal pain, nausea, and vomiting, initially managed at a village hospital. There, a stomach wash with coconut oil was performed seven hours post-ingestion. Acute management was done at the local hospital, but no records were available. Consequently, he was referred to Hayatabad Medical Complex for further evaluation due to jaundice and anemia.

Upon admission, the patient exhibited abdominal pain, nausea, anemia, and yellow discoloration of the skin. Despite denying any prior medical illness or history of anemia, and having no previous hospitalizations, he presented with pallor and intermittent yellow discoloration. Furthermore, there was no family history of G6PD deficiency. On examination, he displayed a pale complexion and scleral icterus, with unremarkable findings upon examination of the abdominal, respiratory, cardiovascular, and central nervous systems. Following the initial assessment, the patient was admitted for observation and further investigation of his anemia and jaundice.

Laboratory investigations during his hospital stay (Table 1) revealed unconjugated hyperbilirubinemia with normal synthetic liver function and serum electrolytes. Peripheral smear analysis demonstrated normocytic hypochromic anemia with very low hemoglobin levels and an elevated reticulocyte count. Additionally, lactate dehydrogenase (LDH) levels were significantly elevated. Malaria and autoimmune hemolytic anemia were ruled out through negative test results for malaria and both direct and indirect Coombs tests. However, G6PD deficiency was detected on the first day of admission.

**Table 1:** Lab investigations during hospital stay mcL: microliter; g/dL: gram/deciliter; fL: femtoliter; %: percent; mg/dL: milligram per deciliter; IU/L: international units per liter; mEq/L: milliequivalents per liter.

Labs	Reference range	Day 1	Day 2	Day 3	Day 4	Day 5
WBC (x103/mcL)	04-11	18.91	15.32	12.4	8.5	N/A
Hemoglobin (g/dL)	11.5-17.5	4.7	5.09	7.09	10	10.8
Mean corpuscular volume (fL)	76-96	82.5	84.4	N/A	N/A	N/A
Platelet count (x103/mcL)	150-450	207	197	N/A	N/A	N/A
Reticulocyte count	0.5-1.5	4	3	N/A	N/A	N/A
Alanine aminotransferase (IU/L)	10-50	24.5	31	25	N/A	N/A
Bilirubin total (mg/dl)	0.1-1.0	9.2	8.4	7.6	4.8	2.8
Bilirubin indirect (mg/dl)	0.1-0.3	8.1	7.3	6.5	3.7	1.3
Alkaline phosphatase (IU/L)	40-129	66.7	75.3	69.1	N/A	N/A
Prothrombin time (seconds)	<12	12	N/A	N/A	N/A	N/A
Activated partial thromboplastin time (seconds)	<34	36	N/A	N/A	N/A	N/A
Lactate dehydrogenase (IU/L)	80-235	2862	2232	1576	970	760
Creatine phosphokinase (IU/L)	5-100	166	130	123	106	91
Urea (mg/dl)	18-45	39	44	N/A	N/A	N/A
Creatinine (mg/dl)	0.64-1.2	1.2	1.01	N/A	N/A	N/A
Na (mEq/L)	35-150	136	133	N/A	N/A	N/A
K (mEq/L)	3.5-5.1	3.9	4.5	N/A	N/A	N/A
Cl (mEq/L)	96-112	99.2	101	N/A	N/A	N/A
Ca ( mg/dl)	08-10	9.1	8.8	N/A	N/A	N/A
Hepatitis B surface antigen (HBsAg)	N/A	Negative	N/A	N/A	N/A	N/A
Anti-hepatitis C virus	N/A	Negative	N/A	N/A	N/A	N/A
Blood phosphine	N/A	Positive	N/A	N/A	N/A	N/A
Malarial parasite	N/A	Negative	N/A	N/A	N/A	N/A
Coombs direct	N/A	Negative	N/A	N/A	N/A	N/A
Coombs indirect	N/A	Negative	N/A	N/A	N/A	N/A
G6PD (minutes)	N/A	75	N/A	N/A	N/A	N/A

Imaging studies, including ultrasound of the abdomen and pelvis, echocardiogram (ECG), and chest X-ray, were all within normal limits. Treatment included IV hydration, a stat dose of vitamin E 600 mg, followed by administration every four hours up to 17 doses, and MgSO4 1 gram stat, followed by 1 gram every six hours for five days, alongside symptomatic management.

Due to a decrease in hemoglobin levels, the patient received a transfusion of three pints of packed red blood cells on the second day of admission. Following clinical and laboratory improvement, he was discharged on the fifth day. A referral to the psychiatry department for depression treatment was made, and advice was provided regarding G6PD deficiency, along with recommendations on avoiding triggers for future anemia and jaundice episodes. Subsequent testing after two months confirmed persistent G6PD deficiency.

## Discussion

Glucose-6-phosphate dehydrogenase (G6PD) deficiency is a hereditary condition inherited in an X-linked recessive manner. It is characterized by decreased levels of G6PD, an enzyme crucial in the pentose phosphate pathway, particularly significant in red blood cell metabolism. This deficiency represents the most prevalent enzymatic disorder affecting red blood cells in humans. G6PD deficiency leads to the sudden destruction of red blood cells upon exposure to various triggers such as fava beans, certain medications, toxins, poisons, and metabolic abnormalities. While most individuals with G6PD deficiency do not display symptoms, symptomatic cases are predominantly observed in males due to the X-linked inheritance pattern. However, female carriers may also experience clinical manifestations due to random inactivation of an X-chromosome in certain cells, resulting in a mixture of G6PD-deficient and normal red blood cells. The abnormal breakdown of red blood cells in G6PD deficiency can present in different ways, including prolonged neonatal jaundice, which could potentially lead to kernicterus - an extremely severe complication. A hemolytic crisis may occur in response to various factors such as infections, certain medications (like anti-malarial, sulfonamides, thiazolesulfone, methylene blue, and specific analgesics), certain foods (especially broad beans), certain chemicals, and diabetic ketoacidosis [3].

G6PD deficiency confers protection against falciparum malaria due to the susceptibility of the parasites to the oxidative stress caused by G6PD deficiency. It has been proposed that the parasite utilizes the nicotinamide adenine dinucleotide phosphate (NADPH) present in the red blood cells (RBCs) of the host to maintain its own glutathione in a reduced state. Consequently, it was theorized that individuals with G6PD deficiency might be less susceptible to severe malaria infection, as the simultaneous utilization of NADPH by both the host erythrocyte and the malaria parasite could exceed the limited capacity of G6PD-deficient red cells to regenerate NADPH. This would result in a depletion of glutathione (GSH) and subsequent oxidative hemolysis. Indeed, it appears that the oxidative stress induced by a toxin in G6PD-deficient individuals leads to the lysis of RBCs, thus hindering the further spread of the parasites. Oxidation of the host RBCs prevents the parasite from maintaining its own glutathione in a reduced state, ultimately causing damage to the parasite [4,5].

The concept of oxidative stress and the release of free oxygen radicals outside of the mitochondria have been proposed as potential mechanisms underlying ALP poisoning and fatalities [6]. It appears that by mitigating the oxidative stress induced by phosphine gas, we may be able to rescue the patient. Due to the significant lysis of RBCs in individuals with G6PD deficiency, a hypothesis has emerged suggesting the potential prevention of the widespread distribution of oxidative stress by the phosphine-damaged RBCs [7]. In the literature, four cases of ALP poisoning in G6PD-deficient individuals who survived have been described. One such case was documented in 2015 in Pakistan, where a 15-year-old male patient survived despite experiencing unconjugated hyperbilirubinemia resulting from hemolysis [8]. A 24-year-old with G6PD deficiency ingested one ALP tablet and was managed conservatively. After two days, they returned with jaundice and hemoglobinuria but improved and were discharged symptom-free the following day. Another case involved a 22-year-old with G6PD deficiency who ingested one ALP tablet, presenting with nausea and vomiting. They were managed conservatively and discharged upon improvement [9,10].

To explore the protective role of G6PD deficiency, a study was carried out in Iran using rat hepatocytes. G6PD deficiency was induced in these cells using 6-aminonicotinamide (6-AN). The results showed that G6PD deficiency markedly decreased the hepatotoxic effects of ALP in rats. This suggests that drugs inducing G6PD deficiency in humans may offer protection against ALP poisoning [11].

Although there is no targeted therapy for ALP poisoning, both experimental and clinical investigations have suggested that various substances such as magnesium sulfate, melatonin, N-acetylcysteine, glutathione, sodium selenite, vitamin C, vitamin E, triiodothyronine, liothyronine, vasopressin, milrinone, Laurus nobilis L., 6-AN, boric acid, acetyl-L-carnitine, and coconut oil could potentially act as antidotes. These substances may mitigate the harmful oxidative effects of ALP [12].

## Conclusions

In conclusion, ALP poisoning remains a significant public health concern, with high mortality rates and limited treatment options. The rise in cases presenting to medical facilities underscores the urgent need for effective interventions. Our case presentation highlights the potential protective role of G6PD deficiency against the complications of ALP poisoning, suggesting avenues for further research into novel therapeutic strategies. Additionally, the experimental evidence from rat hepatocytes underscores the importance of exploring pharmacological interventions that induce G6PD deficiency to mitigate the hepatotoxic effects of ALP. While current treatment options are limited, various substances have shown promise as potential antidotes by reducing the oxidative stress induced by ALP. Further studies are warranted to elucidate the underlying mechanisms and to develop targeted therapies for ALP poisoning. Overall, this case underscores the importance of continued research and vigilance in addressing this challenging clinical problem.
